# Measuring the similarity of charts in graphical statistics

**DOI:** 10.1038/s41598-024-56156-5

**Published:** 2024-03-22

**Authors:** Krzysztof Górnisiewicz, Zbigniew Palka, Waldemar Ratajczak

**Affiliations:** 1 Faculty of Mathematics and Computer Science, Adam Mickiewicz University, Poznan, Poland; 2 Faculty of Human Geography and Planning, Adam Mickiewicz University, Poznan, Poland

**Keywords:** Graphical statistics, Figures, Similarity, Metrics, Spatial analysis, Neutrosophic numbers, Applied mathematics, Software, Statistics, Applied mathematics, Software, Statistics

## Abstract

Figures used in statistics and other sciences play a vital role in understanding and analyzing the problems under study. Due to the complexity and diversity of these problems, figures such as cartograms, choropleth maps, or radar charts take various geometric forms. Their visual evaluation from the view of geometric similarity is essential but insufficient. This paper proposes and theoretically justifies new metrics based on graph theory. They make it possible to quickly determine the degree of similarity of the statistical figures used in the research procedure. The new metrics were used to 1. Determine the similarity of the domestic route networks of major U.S. airlines, 2. Determine the similarity of the distribution of votes cast in U.S. presidential election in each state in 2016 and 2020, 3. Compare radar charts of some countries, constructed based on the Global Competitiveness Index, 4. Analyze the similarity of neutrosophic double line graphs representing sets of approximate (neutrosophic) numbers. This improves analytical capabilities concerning various processes mapped with well-known types of statistical charts, such as choropleth maps, radar charts, etc.

## Introduction

Graphical statistics provides ample opportunities not only to describe, but also to better understand a range of processes—natural, geographic and social.

Having originated in the 15th century^1^ and made significant advances in the 18th and 19th^2–6^, the field is in constant development. It has seen particular progress since the emergence of graphics and computer cartography; see^7–15^.

The historical development of graphical statistics is presented in depth in seminal papers by authors such as Friendly and Tobler.

Researchers’ scientific needs and creative capabilities have resulted in using many types of geometric charts in graphical statistics, depicting various properties and interdependencies of different natural and social processes. Some have played and continue to play critical roles in analysis in statistical, econometric, economic, and geographical studies and in many other sciences. Significant types include choropleth maps, cartograms, and radar charts. Their valuable property is that they can also be represented as a graph.

Visual assessment is the primary way to determine the degree of similarity of such charts corresponding to the spatial units under study. For example, one may assess the similarity of their shape as substantial, average, weak, etc. Then, one may use a nominal or ordinal scale of measurement. This limits the possibility of applying certain mathematical operations. On the other hand, the use in such a case of an interval or quotient scale requires the definition of an appropriate metric, enabling an accurate determination of the degree of similarity of the charts. Such an approach is proposed in this work.

We present here a new proposal for determining the degree of similarity between statistical charts of the same type—for example, radar charts—using a newly defined topological metric. The structure of the paper is as follows. The “New metrics between graphical structures” Section presents the construction of the proposed metrics $\varvec{\delta }$, $\varvec{\delta }^*$, $\varvec{\gamma }$, and $\varvec{\gamma }^*$. The metrics are based on the topological properties of statistical graphs. Therefore, some concepts from graph theory are used in this section to the extent necessary to give a strict definition of these metrics. In the “Applications of the new metrics” Section, the defined metrics are used to determine the degree of similarity of well-known types of statistical graphs^16^. These are analyzed in the following areas. First, we compare the structural similarities of three domestic route networks of major U.S. airlines in 2022. Next, we examine the distances between choropleth maps depicting the 2016 and 2020 U.S. presidential election results, including the strength of electoral votes in U.S. states. One of the significant achievements in this paper is the adoption of our metrics for two radar charts instead of two graphs. This allows us to compare the socio-economic situation of the countries depicted in the radar chart. Finally, we perform a similarity analysis of neutrosophic double-line graphs representing sets of approximate numbers.

The work is followed by four appendices containing supplementary materials. Also included is code for quickly determining the degree of similarity of radar charts.

### New metrics between graphical structures

In its simplest form, a network is a collection of points joined together in pairs by lines, which is appropriate here. The points are referred to as vertices and the lines as edges. Many objects of interest in the physical, biological, social, and geographical sciences can be called networks.

Several mathematical models of networks have been implemented (see^17^). Traditional models, such as random graphs and their extensions, mimic the patterns of connections in real networks. The fundamental paper of^18^ initiated essential research on random graphs and their applications, including the contribution of Erdös and Palka’s papers^19,20^. In contrast to the random approach, we will apply here the most basic network model, namely the simple graph introduced by Euler^21^ in 1736.

A simple graph $G=(V, E)$ is a pair of two finite sets, namely a non-empty set of vertices *V* and a set of edges *E*, which is a subset of unordered pairs of vertices from *V*. In particular, the set of edges can be empty; in that case *G* is called a null graph. We will adopt the following labeling convention. In mathematical formulae and inequalities, and only there, the symbol *V* stands for |*V*|—the number of vertices, and *E* stands for |*E*|—the number of edges. This convention allows mathematical formulae to be written in a form that is easier to read and does not cause ambiguity. In graphical statistics, a question naturally arises about the distance between given graphs.

Let us consider two graphs $G_1=(V_1,E_1)$ and $G_2=(V_2,E_2)$. The choice of a metric between these graphs depends on the particular problems under investigation. For example, in a paper by Baláž et al.^22^, issues from organic chemistry were considered. To define the distance between graphs representing chemical structures, they used as a base concept the joint edges of the graphs under consideration, namely

1 $$\begin{aligned} d(G_1,G_2)= E_1+ E_2-2 E^{(1,2)}+ | V_1 - V_2|, \end{aligned}$$

where $E_1, E_2$ are the numbers of edges of graphs $G_1$ and $G_2$, respectively, $E^{(1,2)}$ is the number of common edges in those graphs, and $| V_1- V_2|$ is the absolute value of the difference of the numbers of vertices in those structures. This metric is useful in determining the similarity of graphs in a case when the distribution of edges is important—as in chemical structures.

In applications in geographical and other social sciences, in many cases we are dealing with graphical structures without any connections. In this case, Baláž’s metric (1) is useless, since the absolute value of the difference of the numbers of vertices in such structures does not correctly characterize geographical properties in practical considerations. Furthermore, from a geographical point of view, two subgraphs of a given graph may be treated as identical, even though from the point of view of classical graph theory, those structures are topologically different. To be more precise, in our investigations, two subgraphs representing geographical structures with a common vertex set and the same number of edges will be treated as identical, so the distance between them must be zero. This is not guaranteed by the metric (1).

**Figure 1:**
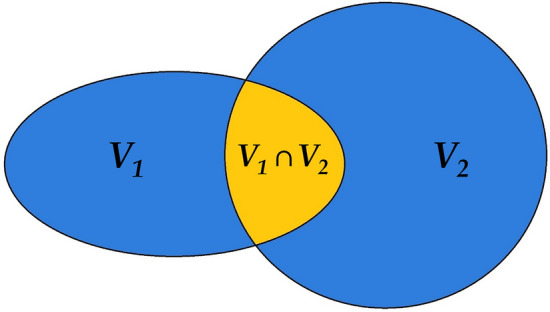
Venn diagram of symmetric difference, $V_1 \triangle V_2$. Source: Own compilation.

Consequently, a new metric, denoted by *PRW*, between graphs $G_1$ and $G_2$ was proposed in a paper by Palka et al.^23^ in which the geographical aspect of the graph is taken into account. A fundamental property of geographic graphs is that their description considers the proper names of the elements of their structure, i.e., edges or vertices. In general, the names describing the vertices of geographic graphs are more important than the names of edges. Here, instead of the notation *PRW*, we will use the Greek letter $\delta$. The primary role in our metric is played by the symmetric difference of the vertex sets $V_1$ and $V_2$ and the absolute value of the difference of the numbers of edges in those structures. The symmetric difference of sets $V_1$ and $V_2$, $V_1 \triangle V_2$, is defined as $(V_1 \cup V_2) \setminus (V_1 \cap V_2)$, and is visualized using a Venn diagram in Fig. 1.

The metric between graphs $G_1$ and $G_2$ is defined by Palka et al.^23^ as follows:

2 $$\begin{aligned} \delta (G_1,G_2)= V^{1\triangle 2}+ | E_1- E_2|, \end{aligned}$$

where $V^{1\triangle 2}$ denotes the number of vertices in the symmetric difference of sets $V_1$ and $V_2$. Note that only two graph parameters determine the value of this metric: the numbers of vertices and edges of the graphs being considered. Furthermore, it is easy to check that $\delta (G_1,G_2)=0$ if and only if $V_1$ is the same as $V_2$ and both graphs have the same number of edges, i.e. $E_1= E_2$, which is consistent with our discussion of the similarity of graphs representing geographical structures.

Since the symmetric difference $V_1 \triangle V_2$ can be expressed as

$$\begin{aligned} (V_1\setminus (V_1\cap V_2)) \cup (V_2\setminus (V_1\cap V_2)), \end{aligned}$$

we have

$$\begin{aligned} V^{1 \triangle 2}= V_1+ V_2-2 V^{(1,2)}, \end{aligned}$$

where $V^{(1,2)}$ stands for the number of common vertices in those graphs. Finally, we obtain our distance in a more convenient form, namely

3 $$\begin{aligned} \delta (G_1,G_2)= V_1+ V_2 -2 V^{(1,2)} + | E_1- E_2|. \end{aligned}$$

The property of symmetry of $\delta$ is obvious, since $\delta (G_1,G_2)=\delta (G_2,G_1).$ Thus, we present a formal proof that for three given graphs $G_1$, $G_2$, and $G_3$, the distance $\delta$ satisfies the triangle inequality, i.e.

$$\begin{aligned} \delta (G_1,G_2)+\delta (G_2,G_3)\ge \delta (G_1,G_3). \end{aligned}$$

Clearly

$$\begin{aligned} | E_1- E_2|+ | E_2- E_3|\ge | E_1- E_3|, \end{aligned}$$

since $|a-b|$ is a metric on the real number line. Thus we need to show only that the following inequality holds:

$$\begin{aligned} V_1+ V_2 -2 V^{(1,2)}+ V_2+ V_3 -2 V^{(2,3)} \ge V_1+ V_3 -2 V^{(1,3)}. \end{aligned}$$

After simple modifications, we obtain the inequality

$$\begin{aligned} V_2 - V^{(1,2)}- V^{(2,3)}+ V^{(1,3)} \ge 0. \end{aligned}$$

It is easy to check that in the case when $V_1\cap V_3$ is the empty set and $V_2$ is contained in $V_1\cup V_3$, the left-hand side of this inequality equals zero. In all other cases, its value is at least one. This completes the proof.

Consequently, the proposed distance between graphs (in the form 2 or 3) satisfies the necessary properties of a metric. In the case of null graphs, this metric will be denoted as $\gamma$ and has the following simple form

4 $$\begin{aligned} \gamma (G_1,G_2)= V_1+ V_2 -2 V^{(1,2)}. \end{aligned}$$

Note that if two graphs are not empty but have the same number of edges, then $\delta =\gamma$. Nevertheless, we will use the notation $\gamma$ only in the case of null graphs.

It terms out that in practical applications, dealing with a relative value of the distance $\delta$ or $\gamma$ is more helpful than their absolute values, as in (3) and (4). Considering the possible applications of the measurement of similarities of geographical subgraphs, we propose in this paper to divide the value of $\delta$ and $\gamma$ by the number of vertices in $V_1\cup V_2$. Consequently, the formulae for the relative distances $\delta ^*$ and $\gamma ^*$ of a given pair of graphs, say $G_1$ and $G_2$, are

5 $$\begin{aligned} \delta ^*(G_1,G_2)= \frac{ V_1+ V_2 -2 V^{(1,2)} + | E_1- E_2|}{ V_1+ V_2 - V^{(1,2)}} \end{aligned}$$

and

6 $$\begin{aligned} \gamma ^*(G_1,G_2)= \frac{ V_1+ V_2 -2 V^{(1,2)}}{ V_1+ V_2 - V^{(1,2)}}, \end{aligned}$$

respectively. The value of the denominator in (5) and (6) is greater than zero, since both $V_1$ and $V_2$ are non-empty sets. As in the case of the metric $\delta$, the relative distance $\delta ^*(G_1,G_2)=0$ if and only if $V_1$ and $V_2$ are the same and $E_1= E_2$. Furthermore, the relative distance for null graphs always satisfies the inequality $0\leqslant \gamma ^* \leqslant 1$.

**Figure 2:**
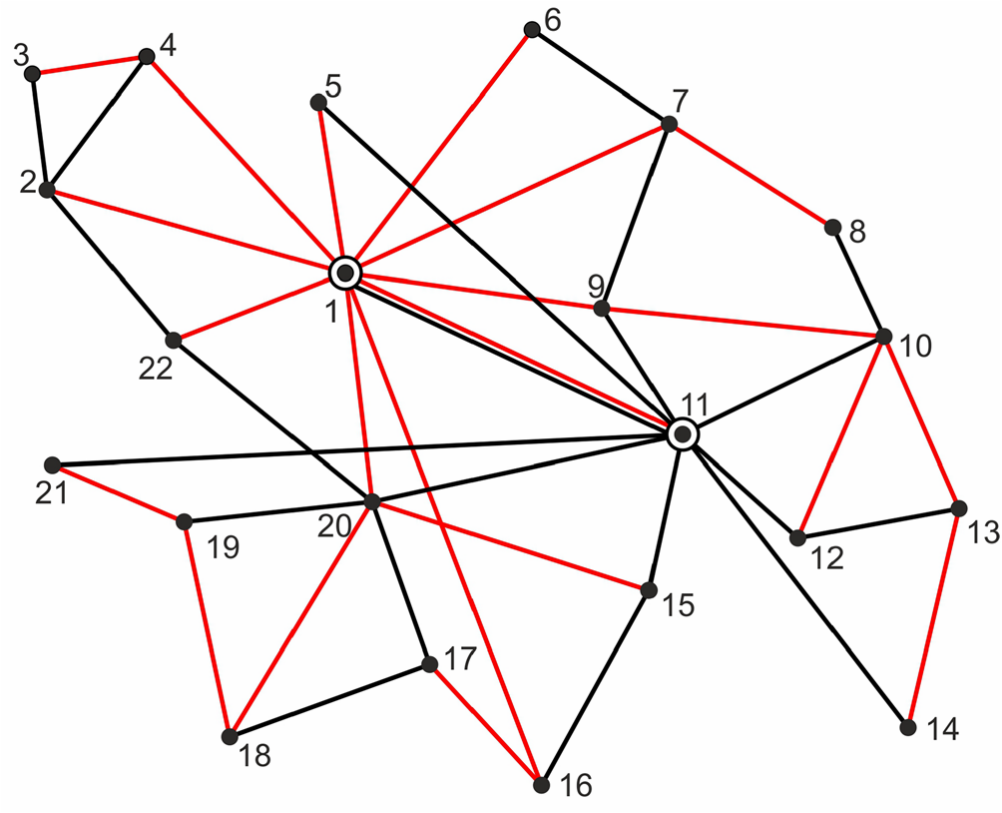
Two subgraphs with the distance $\delta ^*=0$. Source: Own compilation.

Let us emphasize again that the value of the metric $\delta ^*$ is determined by two parameters, the numbers of vertices and edges of the graphs under consideration, and has nothing to do with their topological structures. In Fig. 2, there are two subgraphs (black and red edges, respectively) on the same vertex set $V=\{1,2,\ldots ,22\}$, for which the distance $\delta ^*$ equals zero. This is because both subgraphs have the same number of edges, equal to 21.

A simple transformation of formula (5) provides the following form for our distance:

7 $$\begin{aligned} \delta ^*(G_1,G_2)= 1- \frac{ V^{(1,2)}- | E_1- E_2|}{ V_1+ V_2 - V^{(1,2)}}. \end{aligned}$$

From this formula, it is easy to see that

$$\begin{aligned} \delta ^* < 1\quad\text{if and only if}\quad V^{(1,2)}> | E_1- E_2|. \end{aligned}$$

**Figure 3:**
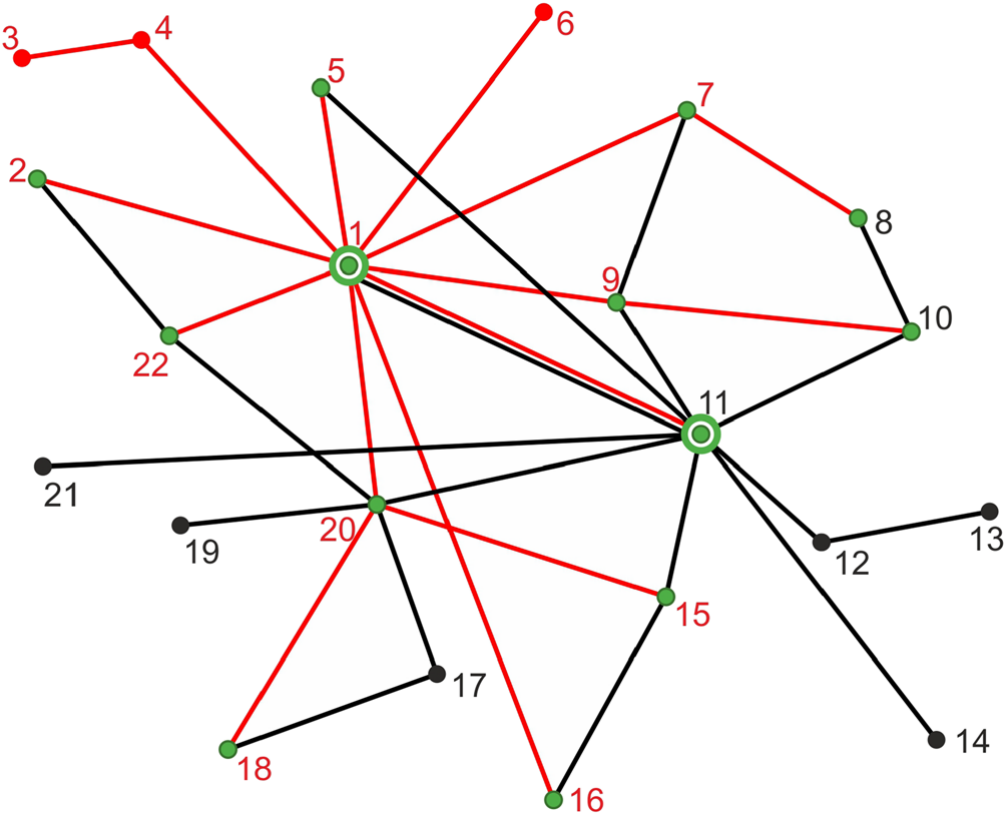
Two graphs with distance $\delta ^*<1$. Source: Own compilation.

To illustrate this case, let us consider the two graphs shown in Fig. 3. The black graph has 19 vertices and 18 edges, whereas the red graph has 16 vertices and 15 edges. Moreover, the two graphs have 13 common vertices (marked green). Consequently the inequality $V^{(1,2)}> | E_1- E_2|$ holds, and by (7) the distance between these graphs is 0.55.

On the other hand

$$\begin{aligned} \delta ^*> 1\quad\text {if and only if}\quad| E_1- E_2| > V^{(1,2)}. \end{aligned}$$

It appears that the value of the relative distance $\delta ^*$ may be substantially large. Indeed, let us consider two graphs $G_1=(V_1,E_1)$ and $G_2=(V_2,E_2)$ where $G_1$ is a complete graph on the vertex set $V_1$, i.e. each pair of vertices from $V_1$ is connected by an edge, and $G_2$ is a null graph having one vertex, which is also an element of $V_1$. Consequently

$$\begin{aligned} E_1= \left( {\begin{array}{c} V_1\\ 2\end{array}}\right) = \frac{1}{2} V_1( V_1-1), \quad V^{(1,2)}=1\;\;\text{and}\;\;V_1+ V_2 - V^{(1,2)}= V_1. \end{aligned}$$

**Figure 4:**
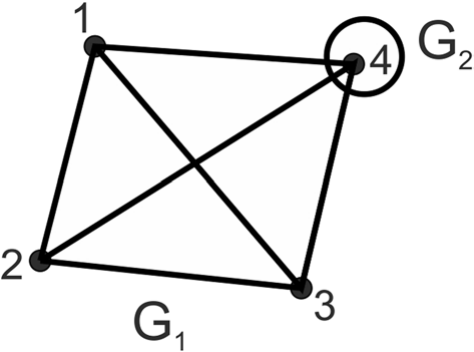
Two graphs for which $\delta ^*>1$. Source: Own compilation.

Thus from (7) we obtain

$$\begin{aligned} \begin{aligned} \delta ^*(G_1,G_2)&= 1+ \frac{| E_1- E_2|- V^{(1,2)}}{ V_1}\\&= \frac{1}{2} V_1 +\frac{1}{2} -\frac{1}{ V_1}\\&\ge \frac{1}{2} V_1, \end{aligned} \end{aligned}$$

if $V_1 \geqslant 2$. To illustrate this case, let us consider the two graphs shown in Fig. 4. $G_1$ is a complete graph on the vertex set $\{1,2,3,4\}$, while $G_2$ is a null graph on a single vertex $\{4\}$. By (7)

$$\begin{aligned} \delta ^*(G_1,G_2) = 2 \frac{1}{4} > \frac{1}{2} V_1 = 2. \end{aligned}$$

**Figure 5:**
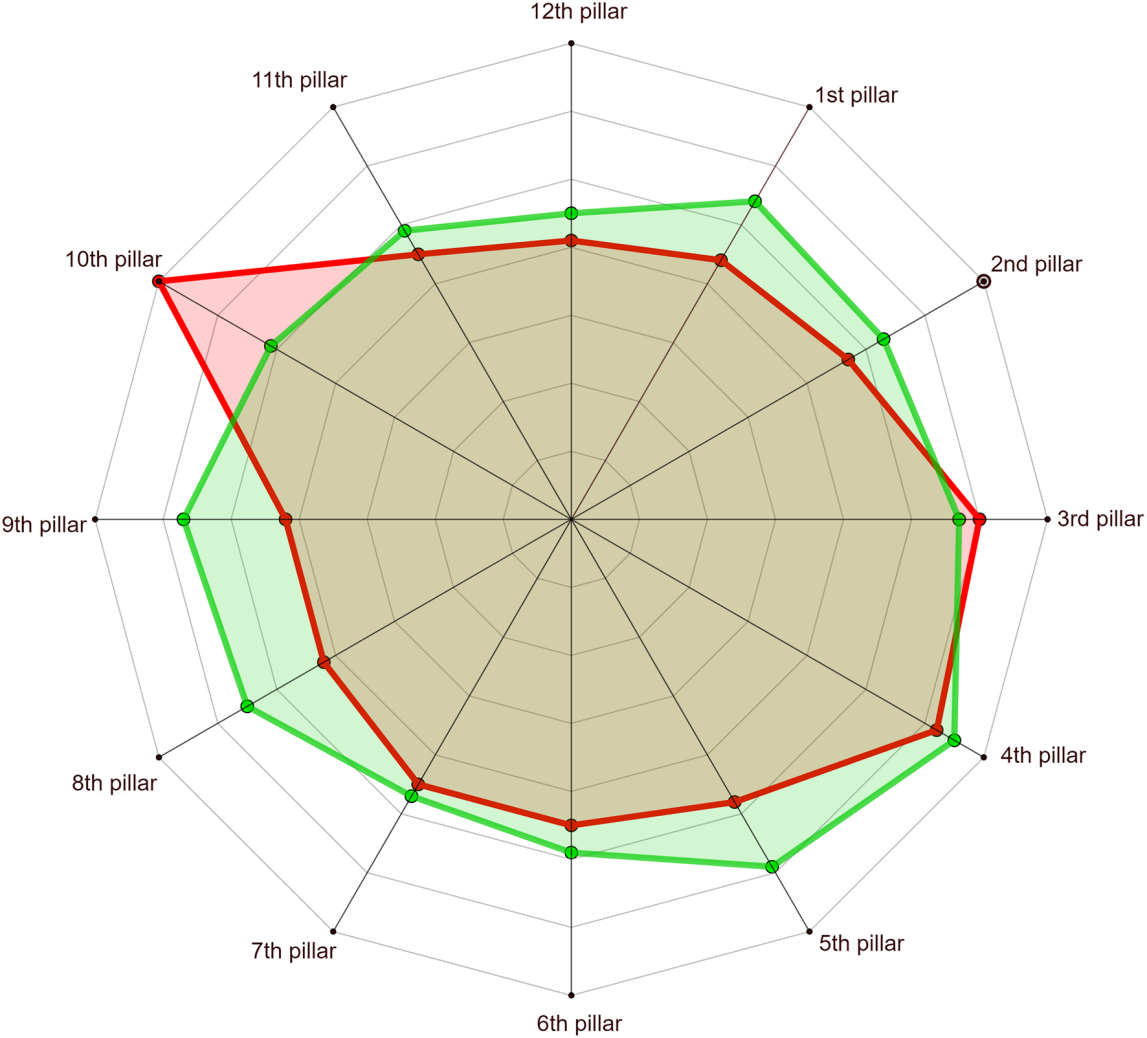
Radar map of two countries. Source: own compilation.

Another task encountered in such fields as international economics, urban economics, socio-economic geography, sociology, etc. is comparison of the socio-economic situation of countries, cities, etc., depicted on a radar chart. This may also be known as a web chart, irregular polygon, star plot polygon, or polar chart. Radar charts have a long history, having been invented by Georg von Mayr in 1877 (see Appendix 2). Figure 5 shows a radar chart of two countries. The image spanned by the values of 100 categories represents an ideal case in the sense that all factors (pillars) are taken into account; for example, some countries are developed to the maximum degree. This is a situation which in reality will probably never occur. However, the question can be posed: what is the distance between specified countries in terms of the given *n* pillars (in the example in Fig. 5, $n=12$)? Here, we propose to adopt a $\gamma$ metric for two radar charts, say $R_1$ and $R_2$, rather than two graphs. Instead of taking into account the number of vertices of the graphs, our metric will be based on the areas of corresponding parts of the radar charts.

Let *A*(*F*) denote the area of a figure *F*. Let $R_{i,1}$ and $R_{i,2}$ denote the *i*-th parts of the given radar charts $R_1$ and $R_2$. Then

8 $$\begin{aligned} \gamma (R_1,R_2) = \sum _{i=1}^n \gamma (R_{i,1},R_{i,2}), \end{aligned}$$

where *n* is the number of pillars in $R_1$ and $R_2$.

First, let us note that the metric $\gamma (R_{i,1},R_{i,2})$ must be considered separately for each *i*-th part of the radar charts. Keeping in mind formula (4) and the assumption that the metric for radar charts is based on the area of corresponding parts, we have, for a given *i*:

9 $$\begin{aligned} \gamma (R_{i,1},R_{i,2})= A(R_{i,1})+A(R_{i,2}) -2A(R_i^{(1,2)}), \end{aligned}$$

where $A(R_{i,1})$ and $A(R_{i,2})$ are the areas of $R_{i,1}$ and $R_{i,2}$, respectively, and $A(R_i^{(1,2)})$ is the area of $R_{i,1} \cap R_{i,2}$. Let $\triangle XYZ$ denote the triangle with vertices *X*, *Y* and *Z*. We have to analyze two significantly different situations.

**Figure 6:**
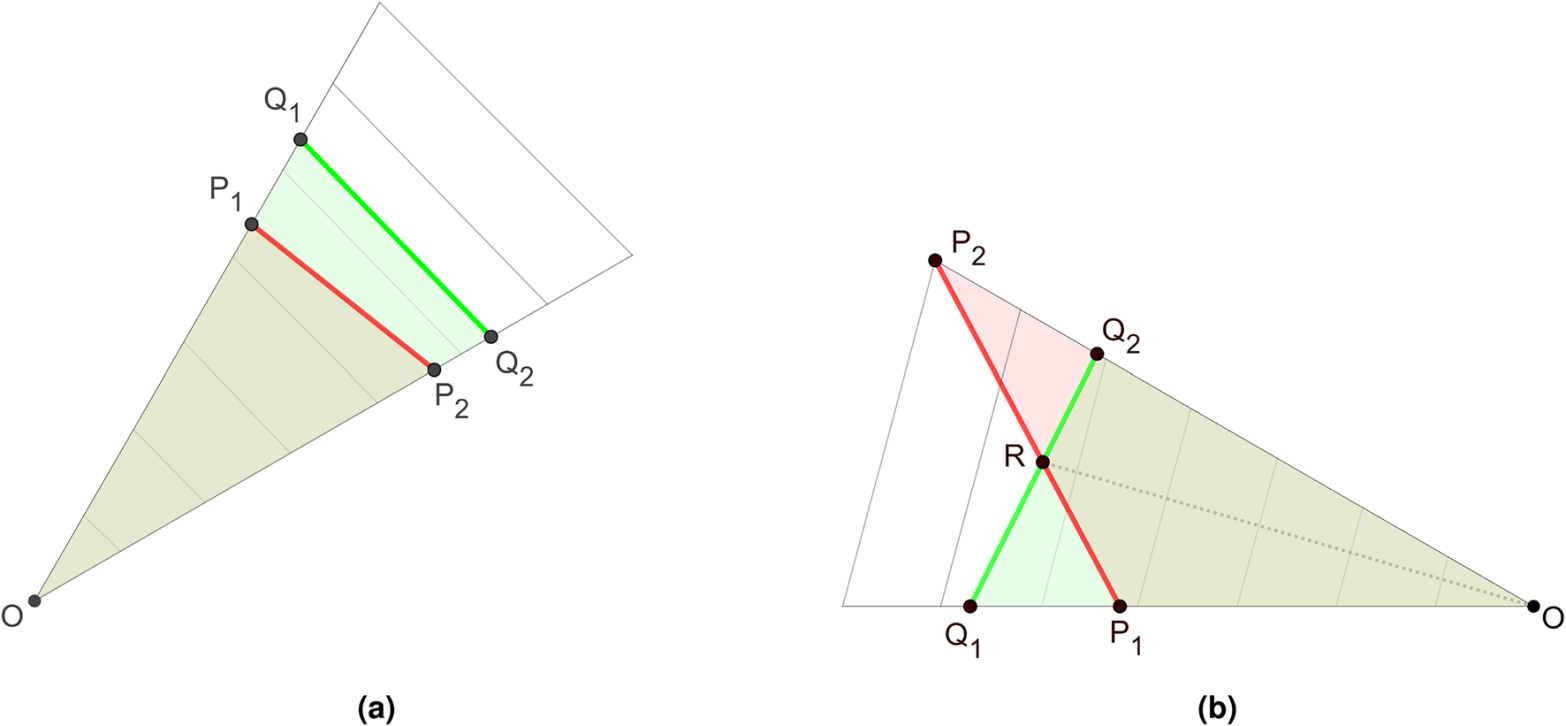
Two different cases of intersection of radar maps. Source: own compilation.

*Case 1*. In a given part the lines of the two tested figures do not intersect.

For example, in Fig. 5, in the part between the first and second pillars, the red line does not cross the green line. This situation is simple to analyze. As shown in Fig. 6a, in this case we have two triangles, say $\triangle Q_1OQ_2$ and $\triangle P_1OP_2$, of which the second is properly contained in the first. Thus, by (9),

10 $$\begin{aligned} \gamma (R_{i,1},R_{i,2})=A(\triangle Q_1OQ_2)-A(\triangle P_1OP_2). \end{aligned}$$

(In practical applications the number of the part of the charts, i.e. the value of *i*, will be known.)

*Case 2*. In a given part the lines of the two tested figures intersect.

For example, in Fig. 5, in the part between the ninth and tenth pillars, the red line crosses the green line. This situation is somewhat more involved to analyze than Case 1. Nevertheless, as is shown in Fig. 6b:

$$\begin{aligned} A(R_i^{(1,2)})=A(\triangle Q_1OQ_2)-A(\triangle P_1RQ_1). \end{aligned}$$

Consequently, by (9)

$$\begin{aligned} \gamma (R_{i,1},R_{i,2})= A(\triangle P_1OP_2 - A(\triangle Q_1OQ_2 ) +2A(\triangle P_1RQ_1) \end{aligned}$$

and finally we have for Case 2, for this particular part of the charts,

11 $$\begin{aligned} \gamma (R_{i,1},R_{i,2})= A(\triangle P_1RQ_1)+A(\triangle P_2RQ_2). \end{aligned}$$

In the case of the metric $\gamma ^*$, let us assume that we are dealing with *m* radars $R_1,R_2,\ldots ,R_m$. Let

$$\begin{aligned} M=\max \left( \left\{ \gamma (R_{i},R_{j}): 1\leqslant i,j\leqslant m\right\} \right) , \end{aligned}$$

be the largest value of metric $\gamma$. Then for a given pair of radars—$R_k,R_l$, say—we define the metric $\gamma ^*$ as follows:

12 $$\begin{aligned} \gamma ^* (R_k,R_l) = \frac{\gamma (R_k,R_l)}{M}. \end{aligned}$$

In socio-economic studies and many others, there are very often situations where available sets of numerical data are ambiguous. Then for example, neutrosophic statistic tools can be used—including neutrosophic statistical graph (see^24–28^). Their spatial structure can be very different. Hence assessing the mutual similarity of such figures can be difficult. The metric derived in this paper make it easy to determinate the degree of similarity between netrosophic graphs.

Based on the determination of the metric $\gamma$ for radar maps, we will now describe an idea of applying our approach to asses the “proximity” of the data represented by uncertain numbers.

In the first step we define a metric between given sets of points on the plane. Let $B=\{(a_1,b_1),\ldots ,(a_n,b_n)\}$ and $C=\{(a_1,c_1),\ldots ,(a_n,c_n)\}$, where $a_1<a_2<\cdots < a_n$, $b_i\geqslant 0$, $c_i \geqslant 0$, be two sets of *n* points. Corresponding to them are the polygons $P_B$, $P_C$ with vertices $P_B=\{(a_1,b_1),\ldots ,(a_n,b_n),(a_n,0),(a_1,0)\}$ and $P_C=\{(a_1,c_1),\ldots ,(a_n,c_n),(a_n,0),(a_1,0)\}$, which define the closed and connected set. Keeping in mind our previous considerations we propose the distance $\gamma (B,C)$ between sets *B* and *C* as

13 $$\begin{aligned} \gamma (B,C)=A(P_B )+A(P_C )-2A(P_B\cap P_C ), \end{aligned}$$

where *A*(*F*) denotes, as before, the area a figure *F* (compare with (10) in the case of radar maps).

**Figure 7:**
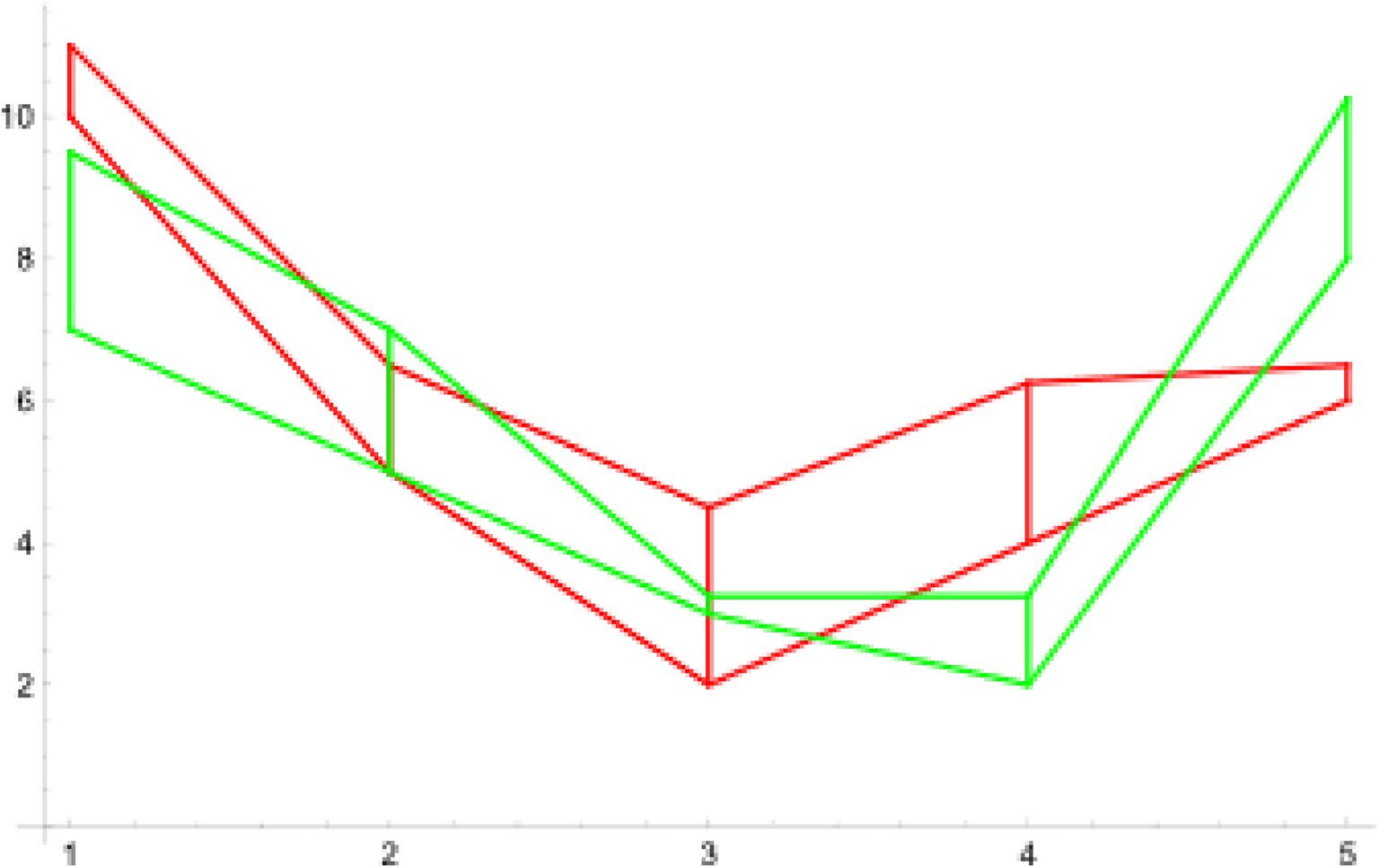
Intersection of two neutrosophic double line graphs ($N_1$-red, $N_2$-green). Source: Own compilation.

Now we ready to define a new metric for neutrosophic sets. Let us assume that we have two data sets of uncertain numbers $N_1=\{d_{11}+u_{11},\ldots ,d_{1n}+u_{1n} \}$ and $N_2=\{d_{21}+u_{21},\ldots ,d_{2n}+u_{2n} \}$ describing *n* given objects (more details about neutrosophic statistic number see^24^). An example of such sets in case when $N_1=\{10+1.0,5+1.5,2+2.5,4+2.25,6+0.5\}$ and $N_2=\{7+2.5,5+2.0,3+0.25,2+1.25,8+2.25\}$, is presented in the form of neutrosophic double line graph on Fig. 7.

Our goal is to propose a metric between $N_1$ and $N_2$, which will be based on a metric between polygons. A crucial point in our considerations is as follows. Instead of using metric (13) directly to the sets $N_1$ and $N_2$, we will consider more sophisticated approach, namely we take into account the minimum and maximum values of uncertain numbers and create the four sets of plane points:

$$\begin{aligned}{} & {} N_1^{\min}=\{(1,d_{11}),\ldots ,(n,d_{1n})\},\\{} & {} N_1^{\max}=\{(1,d_{11}+u_{11}),\ldots ,(n,d_{1n}+u_{1n})\},\\{} & {} N_2^{\min}=\{(,d_{21}),\ldots ,(n,d_{2n})\},\\{} & {} N_2^{\max}=\{(1,d_{21}+u_{21}),\ldots ,(n,d_{2n}+u_{2n})\}. \end{aligned}$$

Consequently having in mind the expression (13) we postulate the following formula for the distance between the sets *X* and *Y* of uncertain numbers:

14 $$\begin{aligned} \gamma (N_1,N_2)=\frac{1}{4} (\gamma (N_1^{\min},N_2^{\min} )+\gamma (N_1^{\min},N_2^{\max} )+\gamma (N_1^{\max},N_2^{\min})+\gamma (N_1^{\max},N_2^{\max})). \end{aligned}$$

**Figure 8:**
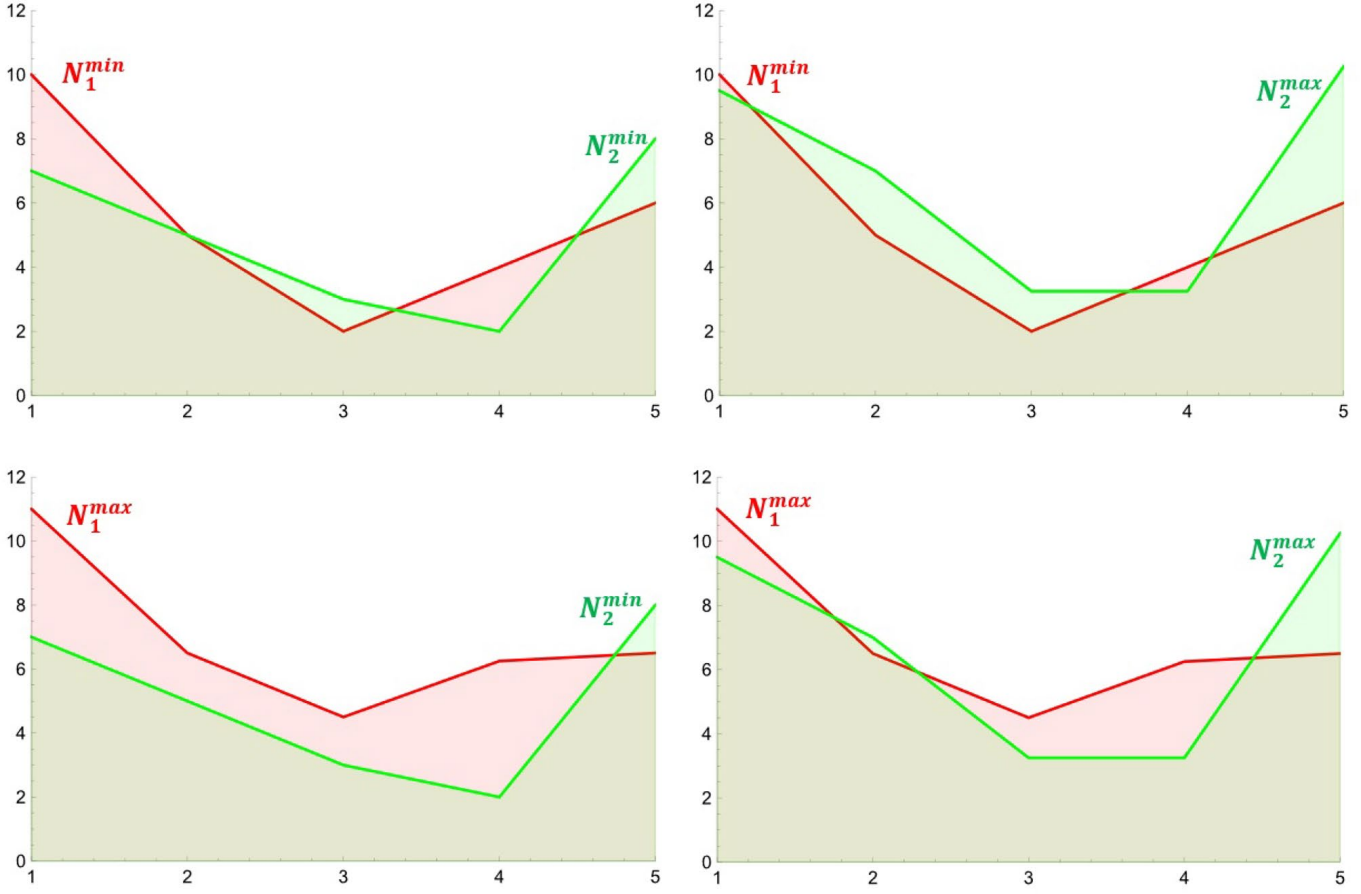
Four pairs of polygons corresponding to neutrosophic numbers $N_1$, $N_2$. Source: own compilation.

To illustrate this idea let us return to the example on Fig. 7; we have the following four sets: $N_1^{\min}=\{10,5,2,4,6\}$, $N_1^{\max}=\{11.0,6.5,4.5,6.25,6.5\}$, $N_2^{\min}=\{7,5,3,2,8\}$, $N_2^{\max}=\{9.5,7.0,3.25,3.25,10.25\}$. Corresponding polygons to these sets are presented on Fig. 8.

The application of newly defined metric $\gamma$ for neutrosohic numbers is outlined in Section “Neutrosophic double line graphs”.

## Applications of the new metrics

### Graphs

**Figure 9:**
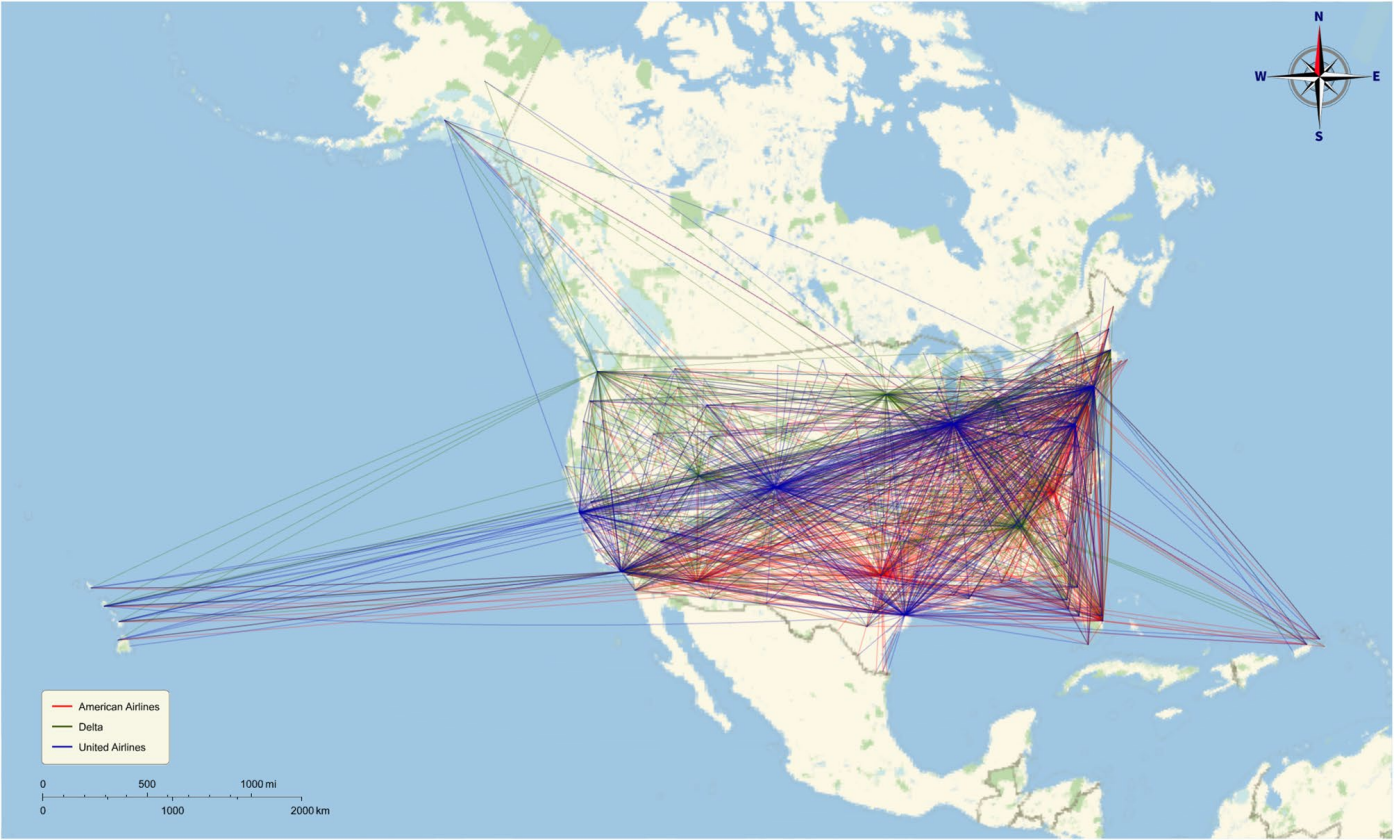
The networks of domestic connections of major U.S. airlines in 2022. Source: Own compilation.

Graphs describe spatial relations using various metrics, often understood as distance functions. They also help determine, for example, the accessibility of certain spatial points, the spatial structure of objects consisting of points and connecting lines, etc. (e.g.^29^). In some scientific work, for example in the procedure of grouping the objects under study due their structural similarity it is necessary to determine the degree of similarity of such objects. The proposed distances $\delta$ and $\delta ^*$ can be used to achieve this goal. We illustrate this by comparing the structural similarities of three major U.S. airlines. It is virtually impossible to determine visually the similarity or dissimilarity of the connection networks of these airlines; see Fig. 9. It is, however, feasible if the $\delta$ and $\delta ^*$ metrics are used.

**Table 1:** Numbers of vertices and edges of the networks of connections of major U.S. airlines and the similarity between them expressed by distance. Source: Authors' calculation.

Airlines	*E*	*V*	$V^{(1,2)}$	$V^{(1,3)}$	$V^{(2,3)}$	$\delta$			$\delta ^*$		
						AA	D	UA	AA	D	UA
1. American Airlines (AA)	898	229	165	162	–	–	380	346	–	1.3869	1.2014
2. Delta (D)	627	210	–	–	154	380	–	174	1.3869	–	0.6282
3. United Airlines (UA)	678	221	–	–	–	346	174	–	1.2014	0.6282	–

Based on the data in Table 1, namely $E, V, V^{(1,2)}, V^{(1,3)}, V^{(2,3)}$, one can easily determine the degree of similarity between the domestic connection networks offered by these airlines. This degree of similarity is determined by the numerical values of the metrics $\delta$ and $\delta ^*$. It can be concluded that in terms of structure, the connection networks of American Airlines and Delta differ the most. On the other hand, the greatest similarity is found between the network structures of Delta and United Airlines. It should be added that the numerical values of the metrics can, of course, be used in various kinds of studies and reports on the spatial optimization of airline connections.

Especially when new air routes are planned and the problem of competition between airlines arises. It should be notes that the metrics used here, can be used to analyze the similarity of the structure of various network like objects.

### Choropleth maps

**Figure 10:**
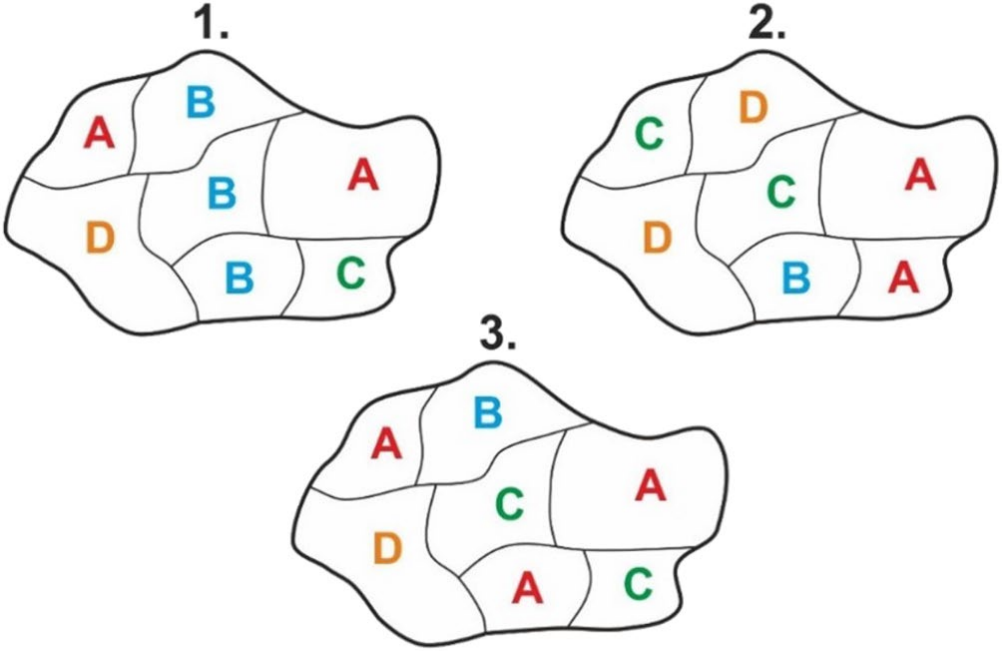
Choropleth maps showing a region whose internal units are classified into different types. Source: Own compilation.

In spatial economics there is often a need to compare various spatial structures, for example, in the form of choropleth maps (see Appendix 2). Figure 10 shows three choropleth maps depicting the same region, whose seven internal spatial units are categorized into four spatial types: A, B, C, and D (In cartography, charts in the form of choropleth maps are also known as cartograms proper, because their scale is discontinuous (discrete).). Comparative analysis requires establishing the similarity between the objects—preferably through an explicitly defined distance. Both $\gamma$ and $\gamma ^*$ can be used for this purpose. It is clear that the regions 1, 2 and 3 in Fig. 10 can be considered as three null graphs with the same number of vertices, namely 7, and different numbers of common vertices. Thus, for example: $\gamma (1,2)=7+7-2\cdot 3=8$, while $\gamma ^* (1,2)=8/11=0.73$. In turn, $\gamma (1,3) = 4$, $\gamma ^* (1,3)=0.44$, $\gamma (2,3) = 8$ and $\gamma ^* (2,3)=0.73$. The result confirms the visual assessment according to which choropleth maps 1 and 3 are the most similar in terms of spatial structure.

**Figure 11:**
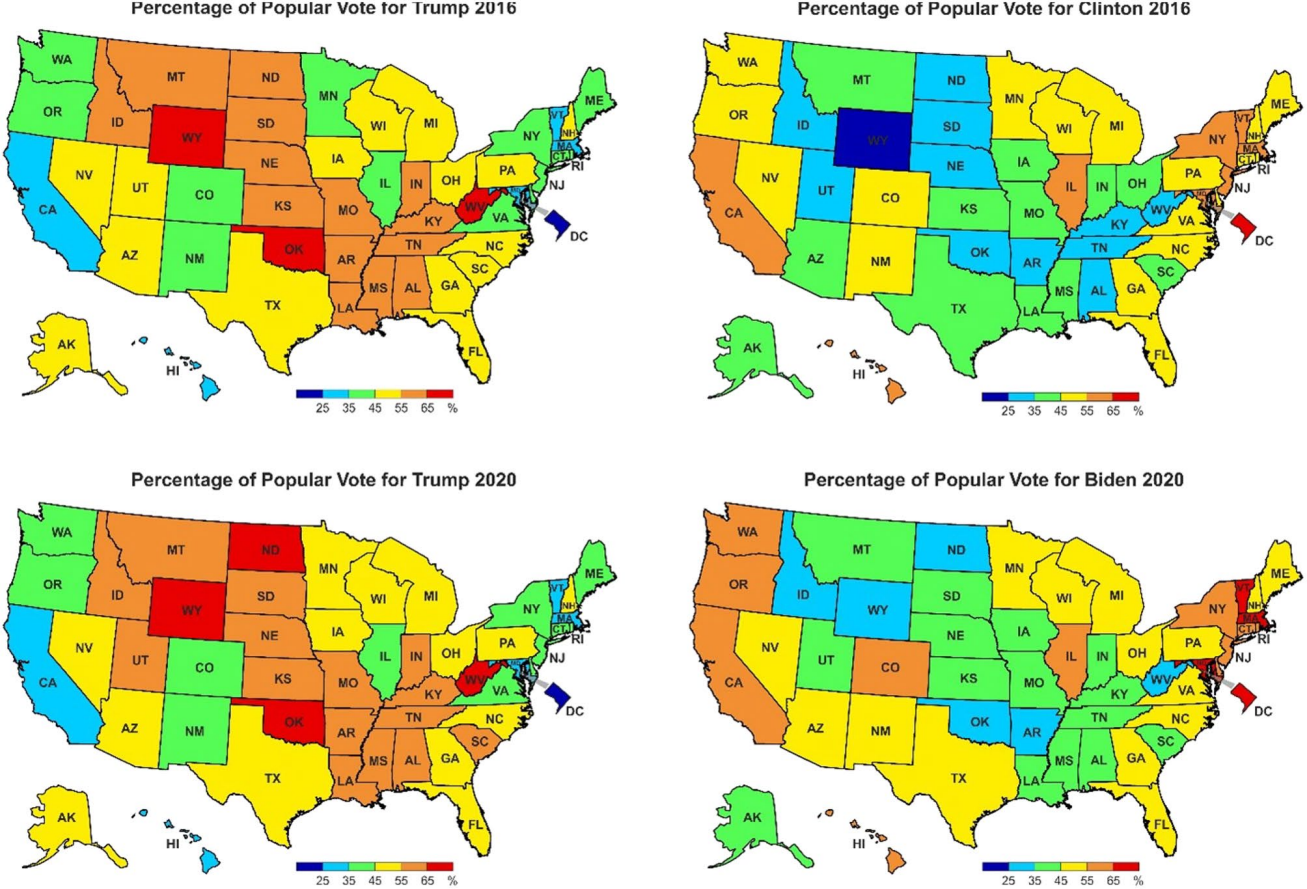
Percentage of popular vote in each state of the USA in 2016 and 2020. Source: Own compilation.

Our next application deals with the 2016 U.S. presidential election, in which the Democratic Party’s candidate was Hilary Clinton and the Republican Party’s candidate was Donald Trump. The choropleth maps in Fig. 11 illustrate numbers of popular votes cast for both candidates. It is easy to see the great spatial variation in these figures, as quantified by the metrics $\gamma$ and $\gamma ^*$ given in Table 2. Thus, it is known that the election results in individual states for the Clinton–Trump contest in 2016 were less similar to each other than for the Biden–Trump contest in 2020. The metrics $\gamma$ and $\gamma ^*$ also enable an extended analysis of the results of the 2016 and 2020 presidential elections. It can be noted, for example, that when the same candidate—Donald Trump, in this case—runs in successive elections, the results obtained by him in individual states in 2020 are not a faithful copy of the results from the previous election, because the values $\gamma =8$ and $\gamma ^*= 0.145$ are very small.

Election analysts can derive many more conclusions based on the values summarized in Tables 2 and 3 or others that can be constructed based on the $\gamma$ and $\gamma ^*$ metrics. Particularly noteworthy, therefore, is the fact that quantification of the differences that occur between analyzed images—here choropleth maps—creates the possibility of further analysis using quantitative methods, which are very important in political and geopolitical analysis, for example.

The results in Table 3 allows us to conclude that:

- The choropleth maps showing the results of voting in each state in 2016 and 2020 for candidate Trump are the most similar. The corresponding values are $\gamma =8$ and $\gamma ^*=0.145$. At the same time, it can be noted that not all states in 2020 voted for candidate Trump as in 2016.
- In contrast, the largest disparity between election results is found for candidates Clinton and Trump in 2016 ($\gamma =84$ and $\gamma ^*=0.93$). It is larger than that between candidates Trump and Biden in 2020 ($\gamma =80$ and $\gamma ^*=0.879$). One can try to determine why?

**Table 2:** Distances between choropleth maps showing the results of the 2016 and 2020 U.S. presidential elections.Source: Own compilation.

Candidates	Metrics	
	$\gamma$	$\gamma ^*$
2016 Clinton–Trump	86	0.915
2020 Trump–Biden	78	0.867

**Table 3:** Distances between choropleth maps showing the results of the 2016 and 2020 U.S. presidential elections for candidates of the same party and candidates of different parties. Source: Own compilation.

Candidates		Metrics	
		$\gamma$	$\gamma ^*$
2016	2020		
Trump	Trump	8	0.145
Clinton	Biden	38	0.543
Clinton	Trump	84	0.903
Trump	Biden	80	0.879

It should be emphasized at this point, that the identification of the degree of similarity between choropleth maps in numerical form creates the possibility of futher in-depth numerical analysis.

### Cartograms

Presidential elections in the U.S. are in fact two-tiered: the President is elected by a college of electors representing each state. Hence, in assessing the influence of individual states on the final outcome of the elections, the electoral strength characterizing each state is an important factor. It can be determined as proposed by^13^ using the formula

15 $$\begin{aligned} {{{Vote\ power}}}=\small {\frac{\quad \,\,\quad \frac{{{Number\ of\ electoral\ votes}}}{{{Number\ of\ popular\ votes}}}}{\text{ mean }\left( \frac{{{Number\ of\ electoral\ votes}}}{{{Number\ of\ popular\ votes}}} \right) }}. \end{aligned}$$

**Figure 12:**
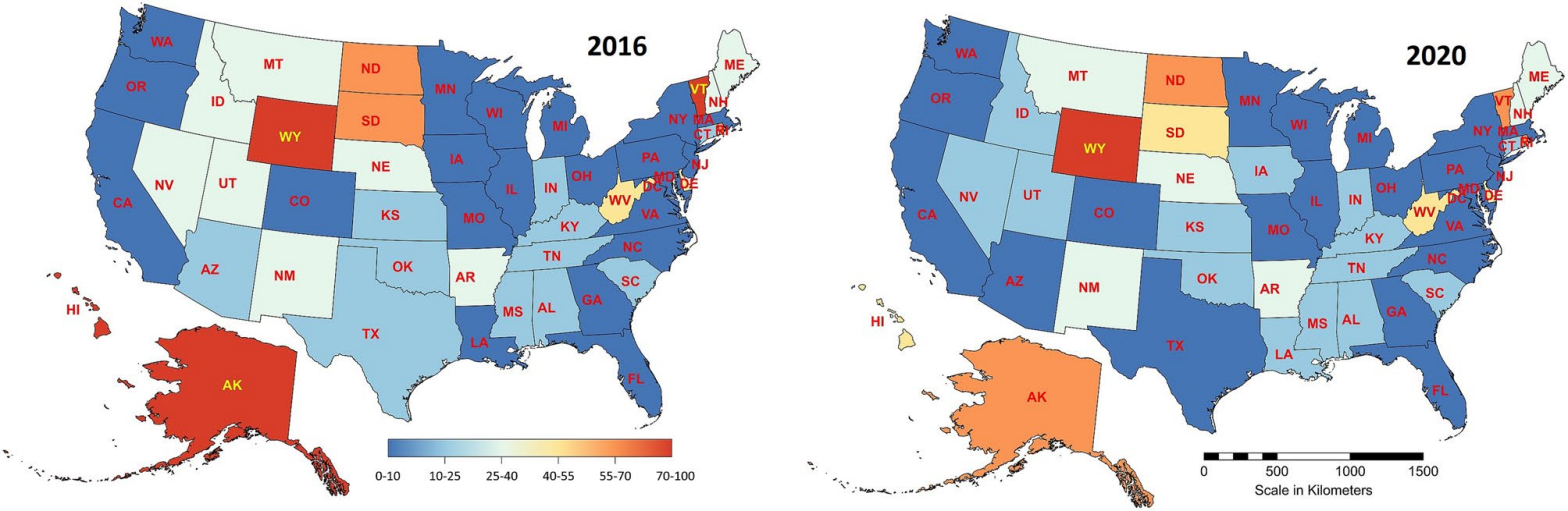
Spatial variability of electoral vote power in the U.S. in the period 2016–2020. Source: own compilation.

The results obtained for the 2016 and 2020 presidential elections are summarized in Table 4. The corresponding cartograms are shown in Fig. 12^30,31^.

**Table 4:** Electoral vote power of U.S. states in 2016 and 2020 presidential elections. Source: Authors' calculation.

No	Code	State	Electoral vote	2016			2020		
				Vote power	Unitized variables	Rank	Vote power	Unitized variables	Rank
1	WY	Wyoming	3	2.2856	100.0	1	2.4168	100.0	1
2	DC	District of Columbia	3	1.8786	75.9	2	1.9424	74.1	2
3	VT	Vermont	3	1.8560	74.5	3	1.8205	67.5	5
4	AK	Alaska	3	1.8353	73.3	4	1.8604	69.6	3
5	HI	Hawaii	4	1.8177	72.2	5	1.5525	52.8	8
6	ND	North Dakota	3	1.6981	65.1	6	1.8476	68.9	4
7	RI	Rhode Island	4	1.6798	64.1	7	1.7225	62.1	6
8	SD	South Dakota	3	1.5800	58.1	8	1.5828	54.5	7
9	WV	West Virginia	5	1.3642	45.3	9	1.4028	44.7	9
10	DE	Delaware	3	1.3176	42.6	10	1.3262	40.5	10
11	NM	New Mexico	5	1.2208	36.8	11	1.2066	34.0	11
12	MT	Montana	3	1.1762	34.2	12	1.1080	28.6	13
13	NE	Nebraska	5	1.1544	32.9	13	1.1657	31.7	12
14	ID	Idaho	4	1.1295	31.4	14	1.0276	24.2	17
15	NH	New Hampshire	4	1.0475	26.5	15	1.1062	28.5	14
16	ME	Maine	4	1.0424	26.2	16	1.0883	27.5	16
17	NV	Nevada	6	1.0392	26.1	17	0.9519	20.1	21
18	AR	Arkansas	6	1.0343	25.8	18	1.0974	28.0	15
19	UT	Utah	6	1.0337	25.7	19	0.8989	17.2	22
20	KS	Kansas	6	0.9874	23.0	20	0.9736	21.2	20
21	MS	Mississippi	6	0.9671	21.8	21	1.0183	23.7	18
22	OK	Oklahoma	7	0.9390	20.1	22	1.0000	22.7	19
23	TN	Tennessee	11	0.8549	15.1	23	0.8031	11.9	28
24	SC	South Carolina	9	0.8342	13.9	24	0.7984	11.7	29
25	AZ	Arizona	11	0.8333	13.8	25	0.7240	7.6	36
26	CT	Connecticut	7	0.8295	13.6	26	0.8557	14.8	24
27	AL	Alabama	9	0.8262	13.4	27	0.8637	15.3	23
28	TX	Texas	38	0.8258	13.4	28	0.7488	9.0	32
29	KY	Kentucky	8	0.8104	12.5	29	0.8348	13.7	25
30	IN	Indiana	11	0.7840	10.9	30	0.8086	12.2	27
31	LA	Louisiana	8	0.7685	10.0	31	0.8304	13.4	26
32	GA	Georgia	16	0.7579	9.4	32	0.7135	7.1	37
33	CA	California	55	0.7559	9.2	33	0.7007	6.4	38
34	IA	Iowa	6	0.7468	8.7	34	0.7912	11.3	30
35	NY	New York	29	0.7321	7.8	35	0.7504	9.1	31
36	WA	Washington	12	0.7052	6.2	36	0.6545	3.8	45
37	NJ	New Jersey	14	0.7044	6.2	37	0.6861	5.6	39
38	IL	Illinois	20	0.7041	6.2	38	0.7391	8.4	33
39	MD	Maryland	10	0.7008	6.0	39	0.7341	8.2	35
40	MO	Missouri	10	0.6940	5.6	40	0.7368	8.3	34
41	OR	Oregon	7	0.6818	4.8	41	0.6573	4.0	44
42	MN	Minnesota	10	0.6619	3.7	42	0.6803	5.2	40
43	WI	Wisconsin	10	0.6549	3.3	43	0.6760	5.0	42
44	MI	Michigan	16	0.6498	3.0	44	0.6440	3.3	47
45	MA	Massachusetts	11	0.6448	2.7	45	0.6754	5.0	43
46	OH	Ohio	18	0.6383	2.3	46	0.6777	5.1	41
47	VA	Virginia	13	0.6359	2.1	47	0.6498	3.6	46
48	PA	Pennsylvania	20	0.6323	1.9	48	0.6428	3.2	48
49	CO	Colorado	9	0.6310	1.8	49	0.6161	1.7	49
50	NC	North Carolina	15	0.6166	1.0	50	0.6053	1.2	50
51	FL	Florida	29	0.6001	0.0	51	0.5842	0.0	51

The indicator (15) is highly dependent on the number of popular votes for each state, which in turn is dependent on the number of residents of the state. Thus, as can be easily seen, the highest electoral vote power is found in such sparsely populated states as Wyoming, Vermont, Alaska, District of Columbia, etc., and the lowest in Florida, North Carolina, Colorado, etc., where the number of residents is large. The $\gamma$ and $\gamma ^*$ metrics help determine the degree of similarity of the cartogram constructed for 2016 to the cartogram for 2020. The numerical values of these metrics are as follows: $\gamma =22$, $\gamma ^*=0.355$. They confirm the relatively high similarity of the two cartograms.

### Radar charts

**Figure 13:**
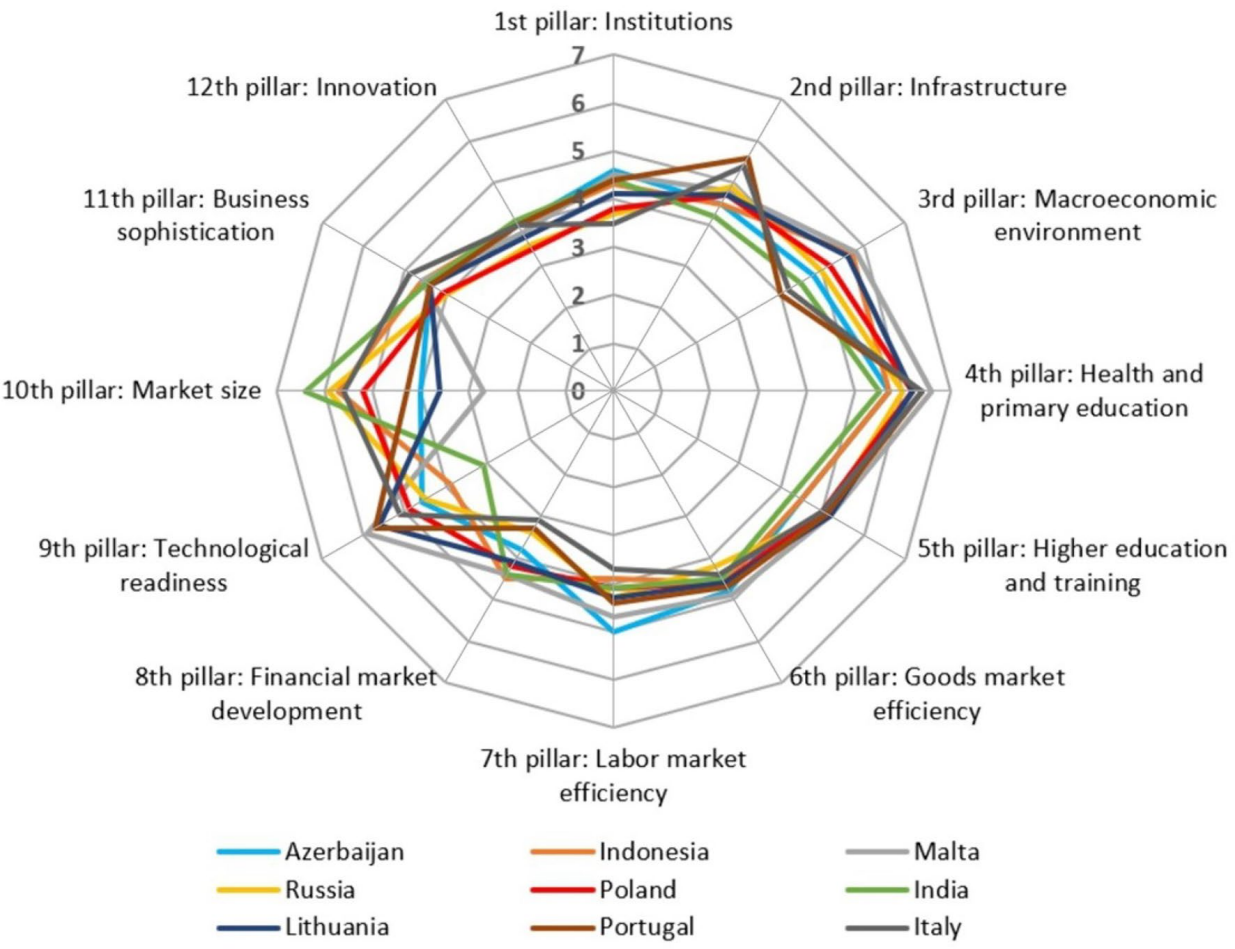
Radar charts of nine countries with similar Global Competitiveness Index values. Source: Authors’ calculations based on WEF 2017–2018 Report.

To illustrate the proposed metrics $\gamma , \gamma ^*$ for establishing the geometrical similarity of radar charts, a set of nine countries with similar values of the competitiveness coefficient (GCI) was selected. These were the countries ranked from 35 to 43, with $4.5\leqslant GCI\leqslant 4.7$ (see WEF 2017–2018). Their radar charts are shown in Fig. 13. The complexity of this figure and the difficult in comparing the different radar charts with each other are readily apparent. Use of the metrics $\gamma$ and $\gamma ^*$ makes it easier to determine the similarity and allows further detailed comparative analysis.

Table 5 includes the above-mentioned information on the nine selected countries. The table also contains the distances between their radar charts in terms of $\gamma ^*$.

**Figure 14:**
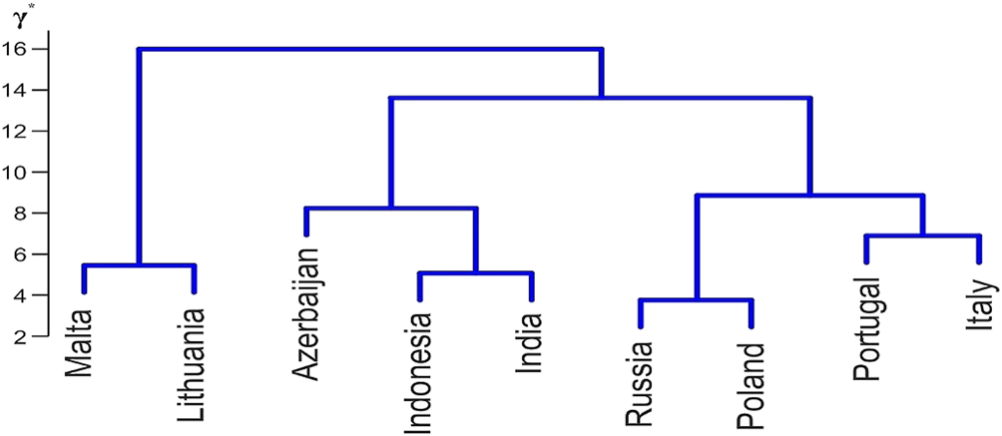
Cluster analysis of nine countries by the Ward method using the $\gamma ^*$ metric. Source: own compilation.

It may be noted that the GCI values suggest dividing the set of countries into only three subsets, i.e. {Azerbaijan, Indonesia}, {Malta, Russian Federation, Poland, India, Lithuania, Portugal}, and {Italy}. In contrast, the numerical values of the metric $\gamma ^*$ used in Ward’s clustering procedure make it possible to divide this set of countries in more detail. This is visualized in Fig. 14. Experience suggests that this division is more in line with the socio-economic situation of these countries. Determination of this division was made possible by the use of $\gamma ^*$.

**Table 5:** Global Competitiveness Index of each country, their ranks, and distances between radar charts. Source: Authors' calculation.

Country	Rank	GCJ	Distances between radar charts								
			1	2	3	4	5	6	7	8	9
1. Azerbaijan	35	4.7	0	0.44056	0.59894	0.56842	0.53254	0.49692	0.47817	0.44521	0.68892
2. Indonesia	36	4.7	0.44056	0	0.75425	0.45504	0.43042	0.31639	0.53313	0.65929	0.55772
3. Malta	37	4.6	0.59894	0.75425	0	0.82855	0.64353	1	0.33696	0.63216	0.94313
4. Russia	38	4.6	0.56842	0.45504	0.82855	0	0.23561	0.58921	0.50699	0.51620	0.35177
5. Poland	39	4.6	0.53254	0.43042	0.64353	0.23561	0	0.64394	0.31459	0.49532	0.46987
6. India	40	4.6	0.49692	0.31639	1	0.58921	0.64394	0	0.78657	0.69274	0.60786
7. Lithuania	41	4.6	0.47817	0.53313	0.33696	0.50699	0.31459	0.78657	0	0.45128	0.66796
8. Portugal	42	4.6	0.44521	0.65929	0.63216	0.51620	0.49532	0.69274	0.45128	0	0.43708
9. Italy	43	4.5	0.68892	0.55772	0.94313	0.35177	0.46987	0.60786	0.66796	0.43708	0

### Neutrosophic double line graphs

Let’s return to the example in Fig. 7, which shows two sets of uncertain numbers, namely $N_1=\{10+1.0,5+1.5,2+2.5,4+2.25,6+0.5\},$ $N_2=\{7+2.5,5+2.0,3+0.25,2+1.25,8+2.25\}$. Using the formula (13), we calculate four metrics $\gamma (N_1^{\min},N_2^{\min})$, $\gamma (N_1^{\min},N_2^{\max})$, $\gamma (N_1^{\max},N_2^{\min})$ and $\gamma (N_1^{\max},N_2^{\max})$, whose values are shown in Table 6 (we used a software system Wolfram Mathematica and build in function, to compute values of intersection areas).

**Table 6:** Values of $\gamma$ metric for pairs of polygons corresponding to $N_1$ and $N_2$. Source: Authors' calculation.

$\gamma (N_1^{\min},N_2^{min})$	$\gamma (N_1^{\min},N_2^{max})$	$\gamma (N_1^{\max},N_2^{min})$	$\gamma (N_1^{\max},N_2^{max})$
3.8333	4.86875	8.8913	4.9762

Finally, according to the proposed formula (14), the distance between the given sets of uncertain numbers $\gamma (N_1,N_2)=5.64239$.

If we have more than two sets of uncertain numbers, using the normalized metric $\gamma ^*$ to compare such numbers is more advantageous. After determining the metric $\gamma$ for each pair of sets of uncertain numbers, we normalize it by the value of the largest of them.

**Figure 15:**
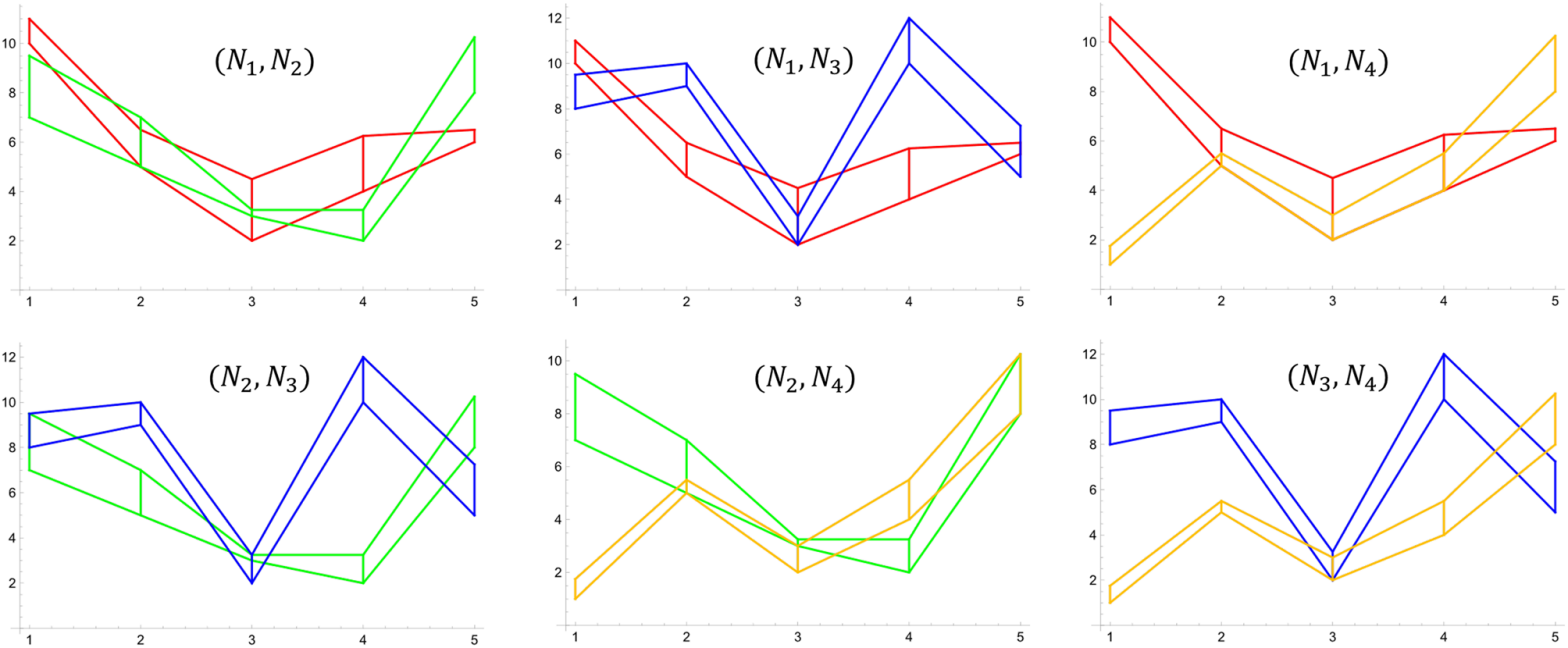
Intersections of six pairs of neutrosophic double line graphs corresponding to four sets of uncertain numbers $N_1$ (red), $N_2$ (green), $N_3$ (blue), $N_4$ (orange). Source: own compilation.

As an example, let us consider two additional sets: $N_3=\{8+1.5,9+1.0,2+1.25,10+2.0,5+2,25\}$ and $N_4=\{1+0.75,5+0.5,2+1.0,4+1.5,8+2.25\}$. Then, we have six possible pairs (see Fig. 15), for which we calculate $\gamma$ metrics. Proceeding as in the example above, we determine areas of polygons for each pair and calculate the $\gamma$ metric, according to formula (14). Then, we normalize each of them by dividing its value by the largest $\gamma$. The relevant results are summarized in Table 7.

**Table 7:** Summary of results. Source: Authors' calculation.

$\gamma (N_1,N_2)$	$\gamma (N_1,N_3)$	$\gamma (N_1,N_4)$	$\gamma (N_2,N_3)$	$\gamma (N_2,N_4)$	$\gamma (N_3,N_4)$
5.64239	9.60552	8.62589	11.5158	7.15514	14.1232
$\gamma ^*(N_1,N_2)$	$\gamma ^*(N_1,N_3)$	$\gamma ^*(N_1,N_4)$	$\gamma ^*(N_2,N_3)$	$\gamma ^*(N_2,N_4)$	$\gamma ^*(N_3,N_4)$
0.399514	0.680126	0.610762	0.815388	0.506625	1.0

In the considered example, the farthest from each other in the sense of our proposed metric are the sets $N_3,N_4$, ($\gamma ^* (N_3,N_4 )=1$), while the closest are the sets $N_1,N_2$ ($\gamma ^* (N_1,N_2 )=0.3995$). This is consistent with the visual assessment of the mutual position of these sets in Fig. 15, but more accurate.

## Conclusions

In the field of statistics, and graphical statistics in particular, many types of chart have been developed to facilitate the understanding and depiction of the relationships occurring in time and space between the various phenomena and factors under study. Some of them are especially frequently used, such as cartograms or choropleth maps. Figures depicting the variability of a phenomenon—for example, over time—show a certain degree of similarity. How can we determine this degree of similarity objectively? This work has provided an answer to that question. The metric $\delta$, constructed by the authors, and its standardized form $\delta ^*$ make it possible to determine the degree of similarity of statistical figures by determining the specific distance between them. In this way, the unavoidable subjectivity associated with the visual evaluation of statistical charts is successfully eliminated—in particular, when the metrics $\gamma$ and $\gamma ^*$ are also used to assess similarity.

This assertion has been confirmed by the empirical analyses carried out in this paper, concerning the similarity of specific graphs, radar charts, choropleth maps and neutrosophic double line graphs that provide geometric representations of studied phenomena.

Also worthy of note is the simplicity of the proposed metrics, and thus the ease with which their numerical values can be calculated.

In many situations it is not necessary to use computers and often expensive software to determine these values. Therefore, we hope that they will prove useful in statistical, economic, geographical, social and other analyses.

### Supplementary Information

Supplementary Information.

## Data Availability

All data used in the article are directly available in the text.
