# Supplementary material for: Measuring the similarity of charts in graphical statistics
Source: Sci Rep. 2024 Mar 22;14:6893. doi: 10.1038/s41598-024-56156-5 (PMC10960004; doi:10.1038/s41598-024-56156-5)
Supplement: Supplementary file 1 — Supplementary Information. [file 41598_2024_56156_MOESM1_ESM.pdf]

Supplementary Material to
Measuring the Similarity of Charts in Graphical Statistics

Appendix 1

Baron Charles Dupin (1784–1873) was a multi-talented French thinker. He made important contributions to such fields as mathematics, economics, education, engineering and politics. In recognition of his great contributions in the field of science, he was elected a member of the French Academy of Science and the Royal Swedish Academy of Science. In his political activity, he became a member of the French Senate. In the field of cartography, he is known as the creator of the first thematic statistical map, showing the distribution of the number of people per school pupil in the 85 departments of France. He constructed this map in 1826 ([1,2]). See the Figure 1, 2 and 3 below.

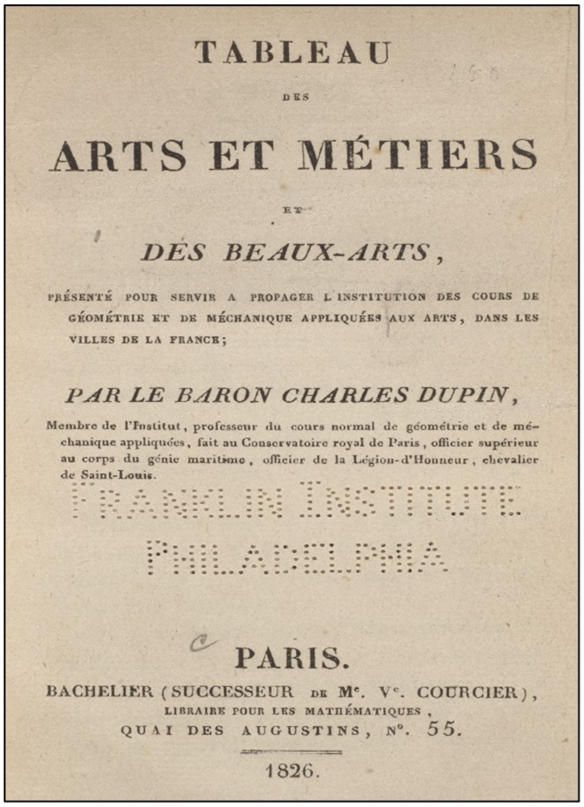

TABLEAU

DES

ARTS ET MÉTIERS

ET

DES BEAUX-ARTS,

PRÉSENTÉ POUR SERVIR A PROPAGER L'INSTITUTION DES COURS DE GÉOMÉTRIE ET DE MÉCHANIQUE APPLIQUÉES AUX ARTS, DANS LES VILLES DE LA FRANCE;

PAR LE BARON CHARLES DUPIN,

Membre de l'Institut, professeur du cours normal de géométrie et de mécanique appliquées, fait au Conservatoire royal de Paris, officier supérieur au corps du génie maritime, officier de la Légion-d'Honneur, chevalier de Saint-Louis.

FRANKLIN INSTITUTE
PHILADELPHIA

PARIS.

BACHELIER (SUCCESSEUR DE Me. Ve. COURCIER),
LIBRAIRE POUR LES MATHÉMATIQUES,
QUAI DES AUGUSTINS, No. 55.

1826.

Figure 1. Work in which Dupin analyzed the various occupations in France at the beginning of the 19th century
Source: https://omeka.lehigh.edu/exhibits/show/data_visualization/case_one/dupin

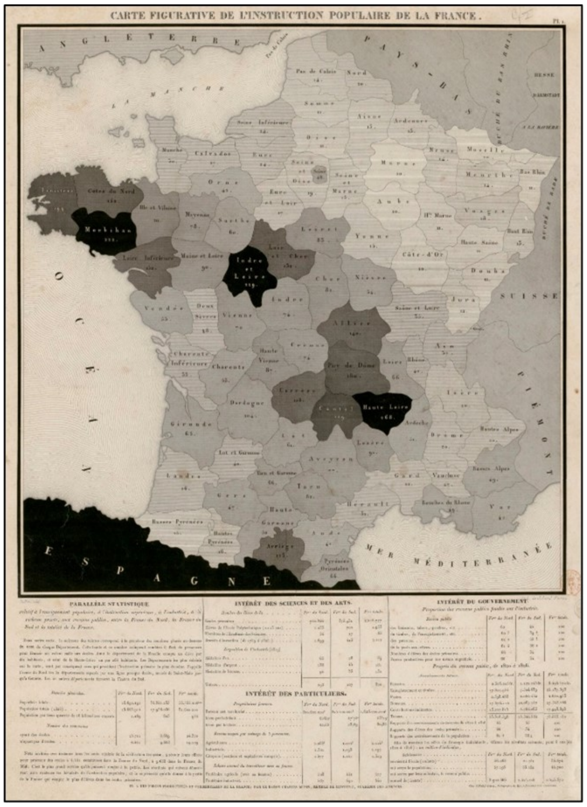

Figure 2. Charles Dupin's thematic map (1826)
Source: https://gallica.bnf.fr/ ark:/12148/btv1b530830640

After more than a century, this type of map was defined by[3] as a "choropleth map." He understood the term as follows: "…the term choropleth, which expresses the idea 'quantity in area,' is tentatively proposed. A choropleth is an areal symbol … [which] indicates densities as actually calculated for the areas that they represent. In the category of choropleth maps would be included maps on which the areas of differing densities are limited by the boundaries of administrative divisions and also maps on which the densities are differentiated within these boundaries" ([3]: 14,[4]: 27).

The choropleth map invented by Dupin immediately became a widely used way to visualize statistical data, and today it is the most common form of representation of cartographic data[2]. Conceptually related to choropleth maps are cartograms. "Cartogram is a map projection that uses purposeful distortion to represent some terrestrial phenomena on a geographic map"[5]. According to[6] and[7], the term "cartogram" was introduced into cartography in 1851 by Charles Joseph Minard (1781–1870), a prominent French civil engineer and cartographer, in the paper[8]. However, this statement may be misleading.

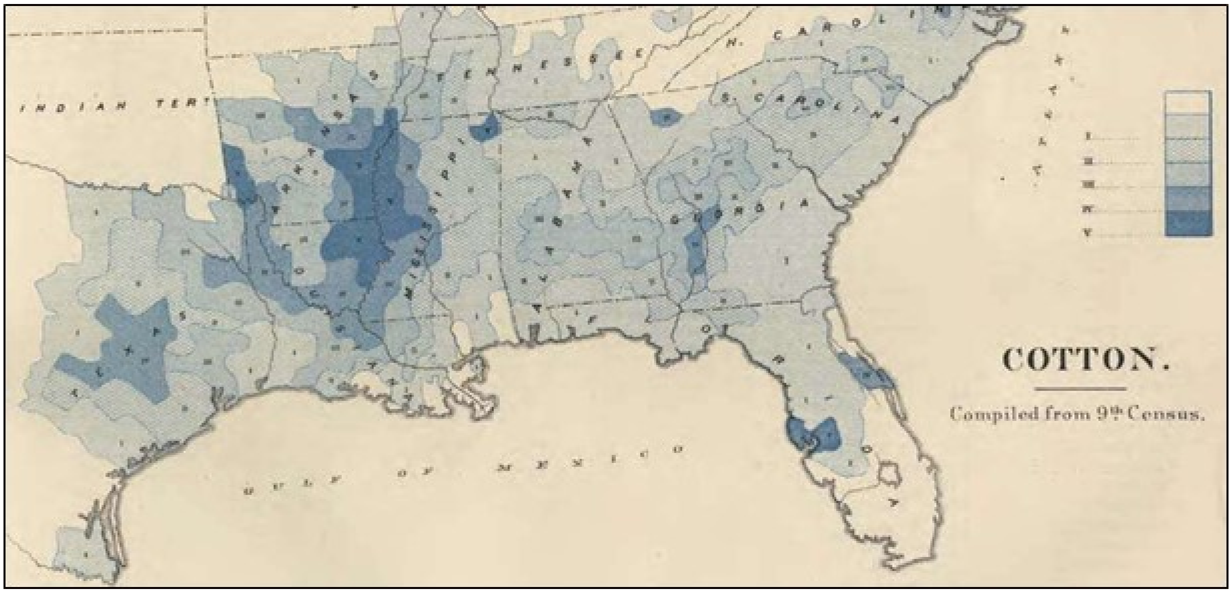

Figure 3. Statistical map (dosimetric cartogram) of 1870 mapping U.S. cotton production
Source: Warf (2010)

A careful analysis of this work shows no evidence of the use of the term "cartogram" by Minard. A similar doubt was expressed by[9]. The first comprehensive review of cartograms of various types was conducted in the work[10]. The very organized and extensive description of cartograms in this work suggests that the term cartogram must have been in use in France earlier. However, no printed sources have been discovered to confirm this supposition. An ingenious classification of cartograms is presented by[7].

Appendix 2

Georg von Mayr (1841–1925) is known in the history of science as the co-inventor of descriptive statistics in the field of administration and international statistics. Despite the fact that his father was a mathematician and an academic lecturer, Georg von Mayr did not advocate the introduction of mathematical statistics into science. Instead, he developed and supported the geometric depiction of statistical relationships in the form of figures and diagrams (Figures 4 and 5).

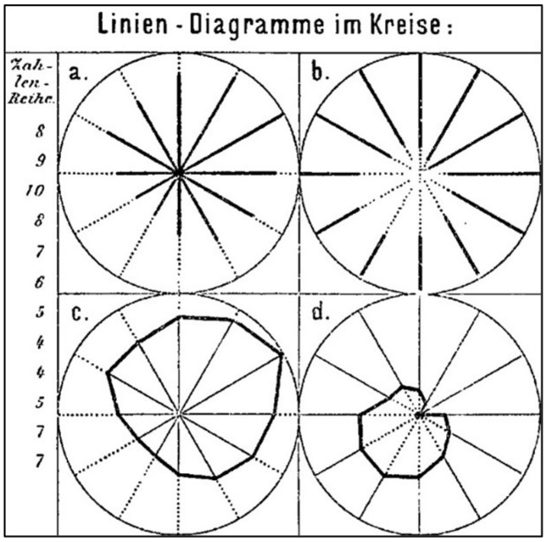

Figure 4. Line graphs within a circle
Source: Mayr G. von (1877). Die Gesetzmäßigkeit im Gesellschaftsleben. Oldenbourg, München, p. 78

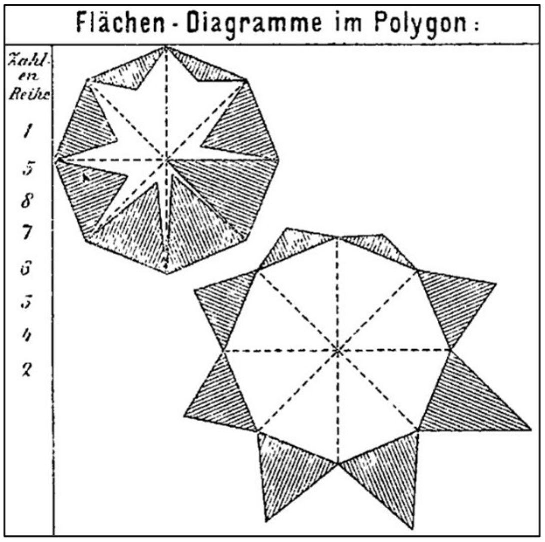

Figure 5. Plane diagrams in a polygon
Source: Mayr G. von (1877). Die Gesetzmäßigkeit im Gesellschaftsleben. Oldenbourg, München, p. 84

He was a tireless organizer of statistical services and institutions in Germany and a member of several statistical societies – including the International Statistical Institute (ISI). He is remembered today as the creator of the radar chart, commonly used to represent – in multidimensional fashion – various properties of objects under study. Figures taken from[11] are shown above.

Appendix 3

Now, we show how to compute the value of metric γ for i-th part of two radar maps for Case 1 and Case 2 presented in 2.4. Assume that, we have the following values of n pillars/categories: $C_1 = (P_1, P_2, \ldots, P_n)$ (radar 1) and $C_2 = (Q_1, Q_2, \ldots, P_n)$ (radar 2). For simplicity, we consider behavior of radar lines for the first pair of pillar values, namely (P_1, P_2) and (Q_1, Q_2)

In Case 1 (see Figure 6a in the main text), the radar lines do not intersect, so by (10 in the main text), metric γ is the difference of two triangle areas and we have the following formula:

$$\gamma_I = \gamma(R_{i,1}, R_{i,2}) = \left| \frac{1}{2} P_1 P_2 \sin\frac{2\pi}{n} - \frac{1}{2} Q_1 Q_2 \sin\frac{2\pi}{n} \right| \tag{1}$$

In Case 2 (see Figure 6b in the main text) we need to find the point R of intersection of the lines P_1P_2 and Q_1Q_2. Basic linear algebra computations give the following coordinates of point R:

$$x(R) = \frac{W_x}{W}, \qquad y(R) = \frac{W_y}{W},$$

where

$$W = \begin{vmatrix} x(P_2) - x(P_1) & y(P_2) - y(P_1) \\ x(Q_2) - x(Q_1) & y(Q_2) - y(Q_1) \end{vmatrix}$$

$$W_x = \begin{vmatrix} x(P_2) - x(P_1) & P \\ x(Q_2) - x(Q_1) & Q \end{vmatrix}$$

$$W_y = \begin{vmatrix} y(P_2) - y(P_1) & P \\ y(Q_2) - y(Q_1) & Q \end{vmatrix}$$

$$P = \begin{vmatrix} x(P_1) & y(P_1) \\ x(P_2) & y(P_2) \end{vmatrix}, \quad Q = \begin{vmatrix} x(Q_1) & y(Q_1) \\ x(Q_2) & y(Q_2) \end{vmatrix}$$

Knowing the location of R, we can determine the value of γ_{II} as the sum of the areas of triangles P_1RQ_1 and P_2RQ_2 (see (11) in the main text). Consequently, in Case 2, the measure of the distance of i-th slices of two radar maps is given by the formula:

$$\begin{aligned}\gamma_{II} &= \gamma(R_{i,1}, R_{i,2}) = \\ &\frac{1}{2} \left\| \begin{matrix} x(R) - x(P_1) & y(R) - y(P_1) \\ x(R) - x(Q_1) & y(R) - y(Q_1) \end{matrix} \right\| + \frac{1}{2} \left\| \begin{matrix} x(R) - x(P_2) & y(R) - y(P_2) \\ x(R) - x(Q_2) & y(R) - y(Q_2) \end{matrix} \right\|,\end{aligned} \tag{2}$$

where $||M||$ means the absolute value of the determinant $|M|$ of matrix M.
To get the total value of the distance $\gamma(G_1, G_2)$ between the radar maps, we need to sum the values of either γ_I or γ_{II} for each of the nth parts.

Appendix 4

Code in C++ used to compute the metric γ^* for k radar charts.

Input data: k – the number of objects (countries, cities, organizations, etc.)
n – the number of categories/pillars for each object
$A[k][n]$ – value tables of categories of each object

Output data: dMax – maximal value of metric γ
matrix of all values of metric γ^*

```
#include <iostream>
#include <cmath>
using namespace std;
int main()
{
   cout«"Number of object:  ";
   int k;
   cin»k;
   cout«"Number of pillars:  ";
   int n;
   cin»n;
   double A[k][n+1];
   for(int i=0;i<k;i++)
   {
      cout«"Values of pillars for object nr "«i+1«":  ";
      for(int j=0;j<n;j++) cin»A[i][j];
      A[i][n]=A[i][0];
   }
   double P[k][n+1][2];
   for(int i=0;i<k;i++)
   {
      for(int j=0;j<=n;j++)
      {
         P[i][j][0]=A[i][j]*cos(2*M_PI*j/n);
         P[i][j][1]=A[i][j]*sin(2*M_PI*j/n);
      }
   }
   double dMax=0.0;
   double G[k][k];    cout«"Table of gamma metrics"«endl;
   for(int s=0;s<k;s++)
   {
      for(int t=0;t<k;t++)
      {
         double d=0;
         for(int j=0;j<n;j++)
         {
            double area;
            if((A[s][j] - A[t][j])*(A[s][j+1] - A[t][j+1]) >= 0.0)
            {
               area=abs((A[s][j]*A[s][j+1]-A[t][j]*A[t][j+1])*sin(2*M_PI/n)/2);
               d=d+area;
            }
            else
            {
               double W, Wx, Wy, Ws, Wt;
               double R[2];
               W=(P[s][j+1][0]-P[s][j][0])*(P[t][j+1][1]-P[t][j][1])
                  -(P[t][j+1][0]-P[t][j][0])*(P[s][j+1][1]-P[s][j][1]);
               Ws=P[s][j][0]*P[s][j+1][1]-P[s][j+1][0]*P[s][j][1];
               Wt=P[t][j][0]*P[t][j+1][1]-P[t][j+1][0]*P[t][j][1];
               Wx=(P[s][j+1][0]-P[s][j][0])*Wt-(P[t][j+1][0]-P[t][j][0])*Ws;
               Wy=(P[s][j+1][1]-P[s][j][1])*Wt-(P[t][j+1][1]-P[t][j][1])*Ws;
               R[0]=Wx/W;
               R[1]=Wy/W;
               area=(abs((R[0]-P[s][j][0])*(R[1]-P[t][j][1])
                  -(R[0]-P[t][j][0])*(R[1]-P[s][j][1]))
                  +abs((R[0]-P[s][j+1][0])*(R[1]-P[t][j+1][1])
                  -(R[0]-P[t][j+1][0])*(R[1]-P[s][j+1][1])))/2;
               d=d+area;
            }
         }
         cout«d«" ";
         if(d>dMax) dMax=d;
         G[s][t]=d;
      }
      cout«endl;
   }
   cout«"Maximal value of metric gamma:  "«dMax«endl;
   cout«"Table of gamma-star metrics"«endl;
   for(int s=0;s<k;s++)
   {
      for(int t=0;t<k;t++) cout«G[s][t]/dMax«" ";
      cout«endl;
   }
   return 0;
}
```

.

References

1. Friendly, M. The golden age of statistical graphics. *Stat. Sci.* **23**, 502–535 (2008).
2. Korycka-Skorupa, J. & Pasławski, J. The beginnings of the choropleth presentation. *Pol. Cartogr. Rev.* **49**, 187 – 198 (2017).
3. Wright, J. K. Problems in population mapping. In Wright, J. K. (ed.) *Notes on statistical mapping, with special reference to the mapping of population phenomena.*, 1–18 (American Geographical Society; Population Association of America, New York, Washington, 1938).
4. Crampton, J. W. Rethinking maps and identity: Choropleth, clines, and biopolitics. In Dodge, M., Kitchin, R. & Perkins, C. (eds.) *Rethinking Maps: New Frontiers in Cartographic Theory*, vol. 11, 26–49 (Routledge, London, 2009).
5. Tobler, W. Cartogram. In Warf, B. (ed.) *Encyclopedia of Human Geography*, 27 (SAGE Publications, 2006).
6. Friis, H. R. Statistical cartography in the united states prior to 1870 and the role of joseph c.g. kennedy and the u.s. census office. *The Am. Cartogr.* **1**, 131–157 (1974).
7. Markowska, A. Cartograms – classification and terminology. *Pol. Cartogr. Rev.* **51**, 51–65 (2019).
8. Minard, C.-J. *Des tableaux graphiques et des cartes figuratives* (impr. de Thunot (Paris), 1862).
9. Tobler, W. Thirty five years of computer cartograms. *Annals Assoc. Am. Geogr.* **94**, 58–73 (2004).
10. Cheysson, E. Les méthodes de statistique graphique à l'exposition universelle de 1878. *J. de la Société Stat. de Paris* **19**, 323–333 (1878).
11. von Mayr, G. *Die gesetzmässigkeit im gesellschaftsleben*. Die Naturkräfte. Eine naturwissenschaftliche Volksbibliothek (Didemburg, 1877).
